# Fast type I interferon response protects astrocytes from flavivirus infection and virus-induced cytopathic effects

**DOI:** 10.1186/s12974-016-0748-7

**Published:** 2016-10-24

**Authors:** Richard Lindqvist, Filip Mundt, Jonathan D. Gilthorpe, Silke Wölfel, Nelson O. Gekara, Andrea Kröger, Anna K. Överby

**Affiliations:** 1 Department of Clinical Microbiology, Virology, Umeå University, 90185 Umeå, Sweden; 2 The Laboratory for Molecular Infection Medicine Sweden (MIMS), 90187 Umeå, Sweden; 3 The Broad Institute of MIT and Harvard, Proteomics and Biomarkers, 415 Main Street, #5033-A, Cambridge, MA 02142 USA; 4 Department of Pharmacology and Clinical Neuroscience, Umeå University, 90187 Umeå, Sweden; 5 Bundeswehr Institute of Microbiology, Neuherbergstraße 11, 80937 Munich, Germany; 6 Department of Molecular Biology, Umeå University, 90187 Umeå, Sweden; 7 Innate Immunity and Infection, Helmholtz Centre for Infection Research, Inhoffen Str 7, 38124 Braunschweig, Germany; 8 Institute of Medical Microbiology, Otto-von-Guericke-University Magdeburg, Leipziger Str. 44, 39120 Magdeburg, Germany

**Keywords:** Astrocytes, Interferon, TBEV, Flavivirus, Viperin

## Abstract

**Background:**

Neurotropic flaviviruses such as tick-borne encephalitis virus (TBEV), Japanese encephalitis virus (JEV), West Nile virus (WNV), and Zika virus (ZIKV) are causative agents of severe brain-related diseases including meningitis, encephalitis, and microcephaly. We have previously shown that local type I interferon response within the central nervous system (CNS) is involved in the protection of mice against tick-borne flavivirus infection. However, the cells responsible for mounting this protective response are not defined.

**Methods:**

Primary astrocytes were isolated from wild-type (WT) and interferon alpha receptor knock out (IFNAR^−/−^) mice and infected with neurotropic flaviviruses. Viral replication and spread, IFN induction and response, and cellular viability were analyzed. Transcriptional levels in primary astrocytes treated with interferon or supernatant from virus-infected cells were analyzed by RNA sequencing and evaluated by different bioinformatics tools.

**Results:**

Here, we show that astrocytes control viral replication of different TBEV strains, JEV, WNV, and ZIKV. In contrast to fibroblast, astrocytes mount a rapid interferon response and restrict viral spread. Furthermore, basal expression levels of key interferon-stimulated genes are high in astrocytes compared to mouse embryonic fibroblasts. Bioinformatic analysis of RNA-sequencing data reveals that astrocytes have established a basal antiviral state which contributes to the rapid viral recognition and upregulation of interferons. The most highly upregulated pathways in neighboring cells were linked to type I interferon response and innate immunity. The restriction in viral growth was dependent on interferon signaling, since loss of the interferon receptor, or its blockade in wild-type cells, resulted in high viral replication and virus-induced cytopathic effects. Astrocyte supernatant from TBEV-infected cells can restrict TBEV growth in astrocytes already 6 h post infection, the effect on neurons is highly reinforced, and astrocyte supernatant from 3 h post infection is already protective.

**Conclusions:**

These findings suggest that the combination of an intrinsic constitutive antiviral response and the fast induction of type I IFN production by astrocytes play an important role in self-protection of astrocytes and suppression of flavivirus replication in the CNS.

**Electronic supplementary material:**

The online version of this article (doi:10.1186/s12974-016-0748-7) contains supplementary material, which is available to authorized users.

## Background

The genus *Flavivirus* belonging to the family *Flaviviridae* include important pathogens causing severe human disease including meningitis, encephalitis, hemorrhagic fevers, and microcephaly. The most significant neurotropic flaviviruses are arthropod-borne tick-borne encephalitis virus (TBEV), West Nile virus (WNV), Japanese encephalitis virus (JEV), and Zika virus (ZIKV). TBEV is transmitted by *Ixodes* ticks, whereas WNV, JEV, and ZIKV are transmitted via mosquitos. No treatments are available for any of these viral infections, and patients are dependent on innate and adaptive parts of the host immune response to fight infections [1–6].

The innate immune system presents a first line of defense against viral infections, for which type I interferons (IFNs) are particularly important. After flavivirus infection, double-stranded RNA (dsRNA) is produced as an intermediate during viral replication. This is sensed as a danger signal in the infected cell by pattern recognition receptors (PRRs) and a signaling cascade is initiated, which leads to the upregulation of IFNs [7, 8]. IFNs are powerful cytokines that mediate antiviral effects via both autocrine and paracrine signaling mechanisms via the IFN alpha receptor (IFNAR). Binding to IFNAR activates the downstream kinases Janus kinase 1 and tyrosine kinase 2, which phosphorylate signal transducer and activator of transcription-1 and transcription-2 (STAT1, STAT2). Together with interferon regulatory factor-9 (IRF9), these form a signaling complex referred to as IFN-stimulated gene factor-3 (ISGF3). ISGF3 translocates to the nucleus and activates the transcription of a large number of interferon-stimulated genes (ISGs) by binding to the interferon response elements. ISGs can inhibit almost every step of a viral life cycle [9, 10].

In mice, the type I IFN response is essential for protection against TBEV, JEV, WNV, and ZIKV infections [11–15]. The CNS has been considered as an immune-privileged tissue; however, recent studies have implicated the importance of intrinsic, innate antiviral responses within the CNS [16–19]. In Langat virus (LGTV, Langat virus, low-virulent member of TBEV serogroup) infection, the local type I IFN response in the CNS has been shown to be critical for the protection of mice against lethal encephalitis [11]. However, the CNS cell type responsible for producing IFN during TBEV infection has not been defined.

While neurons are the main target of neurotropic flaviviruses, other cell types might also become infected and contribute to the resolution of infection [20]. Previous studies have shown that the IFN response and ISG expression in neurons restrict neurotropic flavivirus infection in neurons [19, 21]; however, not much is known about the role of the IFN response in astrocytes during neurotropic flavivirus infection. Recent studies have shown that astrocytes are important IFN-producing cells in various neurotropic viral infections [18, 22, 23]. Astrocytes are one of the most abundant cell types in the brain and mediate diverse supportive functions including ion homeostasis [24, 25], uptake of glutamate [26], free radical scavenging [27], and immune regulation [28]. In TBEV infection, autopsy studies have revealed astrogliosis in post mortem human brains [20, 29], which has been observed for WNV and JEV as well [30, 31]. Indeed, astrocytes have been found to be a site of these infections [32]. However, only a few astrocytes were found to be infected in LGTV-infected mice [33]. They resist infection in an interferon-beta promoter stimulator 1 (IPS-1)-dependent manner and show an activated phenotype, indicating their involvement in LGTV clearance. Both rat and human astrocytes have been shown to be infected in vitro with TBEV; however, the number of infected cells never exceeded 20 %, and the infection did not affect astrocyte viability [34, 35]. Similar findings have also been observed for other neurotropic flaviviruses [36–39]. Therefore, we set out to investigate how the type I IFN system in primary mouse astrocytes contributes to cell survival and restriction of neurotropic flavivirus growth.

We found that astrocytes respond very quickly after viral infection by upregulation of type I IFNs. This upregulation restricts virus replication and spread in primary cultures and contributes to cell survival. By RNA sequencing (RNASeq), we could show that uninfected astrocytes exist in an active antiviral state, which enables fast recognition and response to viral infection by upregulating important antiviral ISGs and that this antiviral state is dependent on IFNAR expression.

## Methods

### Mice

C57BL/6 (wild-type (WT)) mice and IFNAR^−/−^ mice on C57BL/6 background were bred at Umeå Transgene Facility.

### Isolation of astrocytes and neurons

The mice were sacrificed between postnatal day 1 and 4 for astrocyte isolation. Cerebral cortices were isolated, and cells were seeded in poly-d-lysine (Sigma) coated T75 tissue culture flasks as previously described [40]. Monolayers of astrocytes were shaken at 200 rpm for 1 h to remove microglia and oligodendrocyte precursors before seeding for experiments. Primary cortical neurons were derived from cerebral cortices of embryonic day 17 mice. The cortices were isolated, and cells were seeded in Dulbecco’s modified Eagle’s medium (DMEM, Sigma D5648-10L) containing 10 % heat-inactivated foetal bovine serum (FBS, Gibco) and 0.1 U/mL penicillin and 0.1 μg/mL streptomycin (Gibco) in poly-d-lysine (Sigma) coated 96-well plates as previously described [41]. After 3 h, the medium was replaced with Neurobasal medium (Gibco) containing B27 (Gibco), 0.1 U/mL penicillin, 0.1 μg/mL streptomycin, and 2 mM L-glutamine (Gibco). The neurons were infected at day 7 post seeding.

### Viruses and cells

VeroB4 cells were cultured in medium 199/EBSS (HyClone) containing 10 % FBS, 0.1 U/mL penicillin, and 0.1 μg/mL streptomycin (Gibco). Mouse embryonic fibroblasts (MEFs) were grown supplemented with 10 % FBS, 50 μM β-mercaptoethanol (AppliChem), and 2 μg/mL tetracycline DMEM (Sigma). TBEV strains Hypr 71 (isolated in 1953 from blood of a patient in the Czech Republic), Aina (isolated in 1963 in Irkutsk from the blood of a patient), and Sofjin (isolated in 1937 from patient in Russia and showed 99 % sequence identity to strains Sofjin-Chumakov and SofjinKSY with BLAST) were a kind gift of G. Dobler (the Bundeswehr Institute of Microbiology, Munich, Germany). JEV (Nakayama strain) was a kind gift of S. Vene (Folkhälsoinstitutet, Stockholm, Sweden). WNV (isolated in 2003 in Israel WNV_0304h_ISR00, passage number 5) is a kind gift of S. Vene. Stocks were generated in VeroB4 cells. Vesicular stomatitis virus (VSV)-eGFP was propagated in VeroB4 cells. ZIKV MR 766 (isolated in 1947 in Zika Forest, Uganda) was propagated in VeroB4 cells and originally provided by Robert Shope (Yale Arbovirus Research Unit, New Haven, CT, USA) as a reference strain to Jürgen Pilaski (University of Düsseldorf, Germany), who kindly transferred the strain to the Bundeswehr Institute of Microbiology, Munich, Germany. TBEV strains, JEV, and ZIKV are cell culture-adapted reference strains with an unknown passage history. Cell monolayers were infected with virus for 1 h at 37 °C. The virus inoculum was then removed and replaced with DMEM supplemented with 2 % FBS, 0.1 U/mL penicillin, and 0.1 μg/mL streptomycin. Viral titers were determined by focus forming assay as previously described [42].

### RNA isolation and qPCR

Total RNA was isolated at the indicated time points using Nucleospin RNA II kit (Macherey-Nagel), and cDNA was synthesized from 200 to 600 ng RNA as previously described [42]. mRNA expression of GAPDH, IFNβ, IFNα2, viperin and tripartite motif 79α (TRIM79α) were detected by QuantiTect primer assay (Qiagen) and the KAPA SYBR FAST qPCR kit (KAPA Biosystems) using a StepOnePlus fast real-time PCR system (Applied Biosystems). TBEV RNA was quantified using primers previously described [43] and the KAPA PROBE FAST qPCR kit (KAPA Biosystems). Gene expression was normalized to the endogenous GAPDH expression.

### Viral spread assay and immunofluorescence

Cells were grown on 96-well plates (Greiner CELLSTAR®^)^, fixed in 4 % formaldehyde and permeabilized in PBS containing 0.5 % Triton X-100 and 20 mM glycine. The cells were stained with primary antibodies; a mouse monoclonal TBEV anti-E antibody (Hypr, 1493 1:1000 [44], Sofjin and Aina, 1786 1:1000 [44]), flavivirus anti-E antibody (JEV, WNV, and ZIKV, HB112 1:1000, ATCC D1-4G2-4-15 [45]), and rabbit polyclonal anti-GFAP antibody (1:1000 Abcam, ab7260 [46]). Secondary antibodies were as follows (Thermo Fisher Scientific); donkey anti-rabbit Alexa Fluor 488 (A21206), donkey anti-mouse Alexa Fluor 555 (A31570), and goat anti-mouse Alexa Fluor 647 (A21236) which were diluted 1:500. Nuclear counterstaining was performed using DAPI (Life Technologies, D1306) 1 μg/mL. Viral spread was quantified using a TROPHOS Plate RUNNER HD® (TROPHOS SA, Marseille, France). Images of immunofluorescence staining were acquired under a Zeiss Axiovert 25 microscope using an infinity3 luminera® camera.

### IFN bioassay

Supernatants from virus-infected astrocytes were inactivated using β-propiolactone (β-PL) (Acros Organics, 269040050), diluted in water to 0.96 % and used at a final concentration of 0.05 %, incubated at 4 °C for 16 h followed by 2 h incubation at 37 °C for hydrolysis, performed in plates (VWR, 734-2325) to avoid acidification of the samples [47, 48]. The cells (MEFs, astrocytes, or neurons) were seeded in 96-well plates (Greiner CELLSTAR®) and treated with β-PL-inactivated supernatants or serially diluted IFNαB/D [49] (a kind gift of Peter Stäheli, Virology, University of Freiburg, Freiburg, Germany). The cells were infected 24 h post treatment with VSV-eGFP (multiplicity of infection (MOI) 0.01) (MEFs) or TBEV (astrocytes or neurons) and fixed with 4 % formaldehyde at 16 h post infection (hpi). The number of infected cells was determined using a TROPHOS Plate RUNNER HD®, and the IFN levels were quantified using a standard curve (calculated from 1, 5, 10, 50, 100, and 500 U of IFNαB/D).

### Western blot

The cells were lyzed, proteins were separated, and Western blot was performed as previously described [50]. The primary antibodies directed against TBEV E (1493), actin (rabbit polyclonal anti-actin, 1:2500, Sigma [50]), and viperin (mouse monoclonal anti-viperin, 1:500 Abcam [50]) were used.

### RNASeq

Primary WT and IFNAR^−/−^ astrocytes were seeded at 200,000 cells per 12-well. Upon reaching confluence, WT cells were either stimulated with β-PL-inactivated supernatant from WT astrocytes (24 hpi), 5000 U IFNαB/D [49], or left untreated. Six hours after stimulation, total RNA was isolated using Nucleospin RNA II kit (Macherey-Nagel) according to the manufacturers’ instructions. One thousand to three thousand nanograms of RNA was sent to GATC Biotech (Konstanz, Germany) for RNAseq (InView^TM^ Transcriptome Explore).

### Bioinformatics

For analyses of the RNASeq data, all treatments were normalized to their respective control and gene names were converted to human HGNC gene names before bioinformatical analyses were performed. Ingenuity Pathway Analysis (IPA; Ingenuity Systems, http://www.ingenuity.com/) was used to analyze activated pathways and predicted upstream activators as previously described [51, 52]. In brief, activation of *Z* values were used to determine activation of pathway or regulators (−2 ≥ *Z*, significant inhibition; 2 ≤ *Z*, significant activation), *p* values were calculated using right-tailed Fisher’s exact representing the significance for the overlap between dataset and pathway or regulator. Gene set enrichment analysis (GSEA; http://www.broadinstitute.org/gsea) was performed to evaluate functional gene sets that are associated with each of the treatments compared to controls. The fold-change regulation of the genes after treatment was used to perform pre-ranked GSEA. The BIOCARTA, KEGG, and REACTOME databases were queried; *p* values were estimated by 100 permutations and a null-distribution of the enrichment scores. *P* values were adjusted using the Benjamini-Hochberg method. A normalized enrichment score (NES) was computed by mean normalization [53]. The Connectivity Map database (http://www.lincscloud.org/; [54]) was used to analyze upstream regulators based on regulated genes after IFNαB/D or supernatant treatment. The top 50 expressed genes and top 50 downregulated genes were selected from, each treatment, per analysis, and a connectivity score >90 or <−90 was considered significant.

### Blocking of IFNAR receptor

Cells were incubated with DMEM, 10 % heat-inactivated FBS, and 2 mM glutamine with MAR1-5A3 antibody (Affymetrix eBioscience, 16-5945-85 [19]) or IgG1 κ isotype control (eBioscience, 14-4714-85) at a concentration of 10 μg/mL for 2 h at 37 °C. After 2 h, the antibodies were removed, and cells were infected with TBEV for 1 h. Following removal of the viral infection inoculum, the medium containing 10 μg/mL antibody was returned to the cells.

### Resazurin viability test

Three hours before the indicated time point, the cells were treated with 40 μM resazurin; after 3 h of incubation, fluorescent signal was quantified using a plate reader (Paradigm, Beckman Coulter).

### Statistical analyses

Data from quantitative reverse transcription PCR (qPCR), bioassay, focus forming assays, and viral spread assays were analyzed with unpaired *t* test using GraphPad Prism software. Statistical analyses of RNASeq data were performed by GATC Biotech using Cufflinks [55, 56].

## Results

### The type I IFN response limits TBEV replication in astrocytes

**Fig. 1:**
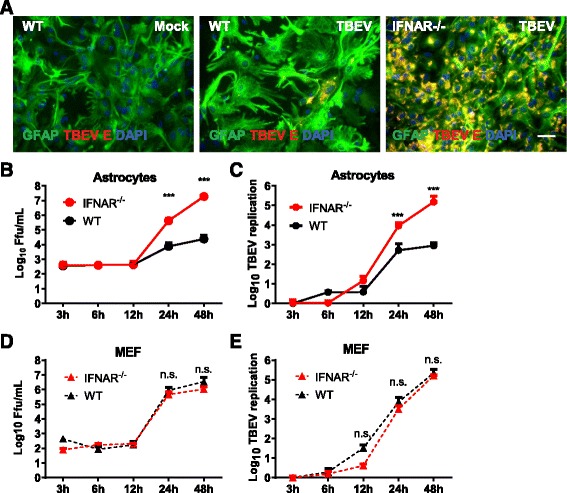
IFNAR restricts TBEV replication in astrocytes. Primary astrocytes were isolated from WT and IFNAR^−/−^ mice and infected with Hypr MOI 0.1. **a** Cell were stained 72 hpi with GFAP (astrocytes), TBEV E protein, and DAPI. Scale bar 193 μm. Primary astrocytes (**b**, **c**) and MEF (**d**, **e**) were infected with TBEV (MOI 0.1), and viral growth was determined over time by focus forming assay (**b**, **d**) and qPCR (**c**, **e**). Data are cumulative from at least two independent experiments performed in triplicates (*n* = 6). *Asterisks* indicate data were statistically significant: ***p < 0.0001

Astrocytes are the most abundant glial cell type in the brain and an important source of type I IFN during various neurotropic viral infections [18, 22, 23]. To investigate the importance of type I IFNs for astrocyte function in neurotropic flavivirus infection, primary astrocytes were isolated from wild-type (WT) and IFNAR^−/−^ mice. These were infected with TBEV strain Hypr at a MOI of 0.1 and analyzed by immunofluorescence (Fig. 1a). Both WT and IFNAR^−/−^ infected astrocytes expressed the characteristic marker glial fibrillary acidic protein (GFAP) and were found positive for TBEV E (Fig. 1a). Viral replication was analyzed over time, and low viral titers and replication were observed in WT astrocytes. In contrast, TBEV titers and RNA replication increased dramatically in IFNAR^−/−^ astrocytes over time (Fig. 1b, c). Infection was also analyzed in MEFs where an unrestricted growth profile was observed in both WT and IFNAR^−/−^ cells (Fig. 1d, e). Our results indicate that IFNAR restricts TBEV replication in a cell type-dependent manner.

### IFNAR restricts neurotropic flavivirus spread in astrocytes

**Fig. 2:**
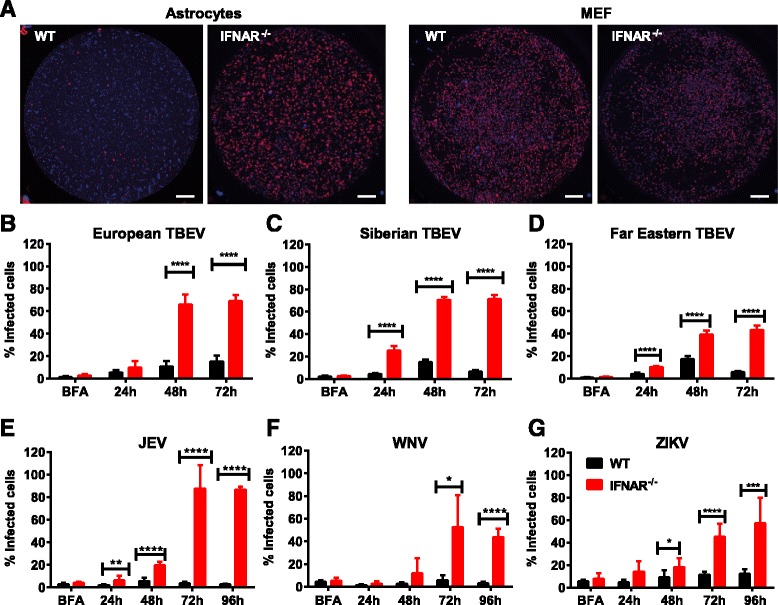
IFNAR restricts neurotropic flavivirus spread in astrocytes. Primary astrocytes isolated from WT and IFNAR^−/−^ mice were infected with 0.1 MOI of different flaviviruses. A number of infected cells were determined by immunofluorescence assay staining for DAPI (*blue*) and flavivirus E protein (*red*). *Scale bar* 0.66 mm. **a** Representative picture of TBEV-infected WT and IFNAR^−/−^ astrocytes and MEFs (resolution 1024 × 1024) 72 hpi. **a**, **b** Hypr European subtype. **c** Aina, Siberian subtype. **d** Sofjin Far Eastern subtype. **e** JEV. **f** WNV. **g** ZIKV. Data are cumulative from at least two independent experiments *n* = 8.*p < 0.05, ***p < 0.001, ****p < 0.0001

The type I IFN response could limit viral replication by different mechanisms: (i) restricting the spread of progeny viruses to neighboring cells, (ii) reducing overall viral replication, or (iii) both. To investigate this, WT and IFNAR^−/−^ astrocytes and MEFs were infected by TBEV at a MOI of 0.1, stained by immunofluorescence, and the numbers of infected cells were quantified (Fig. 2a shows a representative picture of infected wells). Even though the initial infections (24 hpi) in WT and IFNAR^−/−^ astrocytes were comparable, the outcome of infection differed dramatically. Infection of WT astrocytes was low and never exceeded 20 %; however, infection of IFNAR^−/−^ astrocytes was significantly higher at the late time points, reaching an average of 69 % at 72 hpi (Fig. 2b). In MEFs, IFNAR expression failed to restrict TBEV spread, and no difference were observed between WT and IFNAR^−/−^ cells (data not shown). These results suggest that IFNAR restricts the spread of TBEV in astrocytes.

TBEV can be categorized into European, Siberian, and Far Eastern subtypes according to phylogenetic differences. The Siberian and Far Eastern subtypes have been associated with more severe disease compared to the European subtype [57, 58]. Increased pathogenicity could be due to several factors, e.g., ability to interfere with the type I IFN response. Therefore, viral growth of two reference strains; the Siberian Aina and Far Eastern Sofjin, was analyzed in WT and IFNAR^−/−^ astrocytes (Fig. 2c, d). Similar to the European subtype of TBEV, an interferon response also restricted the spread of Siberian and Far Eastern TBEV subtypes (Fig. 2c, d). Although the type I IFN response plays an important role in mosquito-borne neurotropic flavivirus (WNV, JEV, and ZIKV) infections in vivo [12–14, 59, 60], the specific role of IFNAR signaling in restricting viral growth in astrocytes is unknown. WT and IFNAR^−/−^ astrocytes were infected with JEV, WNV, and ZIKV, and the numbers of infected cells were determined (Fig. 2e–g). For all three mosquito-borne viruses, the IFN response was required for the restriction of viral spread in astrocytes. Our data shows that the type I IFN response in astrocytes efficiently restricts the spread of both tick- and mosquito-borne neurotropic flaviviruses.

### Astrocytes induce a rapid IFN response upon TBEV infection

**Fig. 3:**
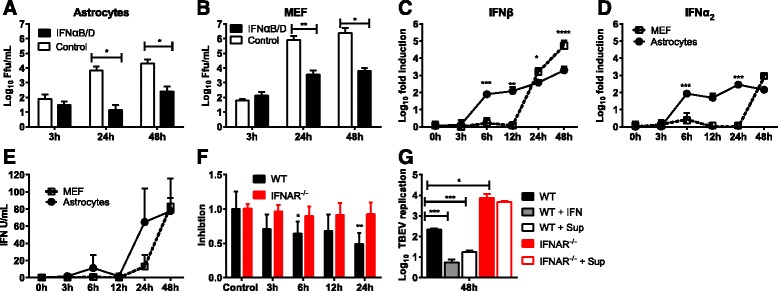
Astrocytes induce a fast type I IFN response upon TBEV infection which restricts virus replication. Primary WT Astrocytes (**a**) and MEFs (**b**) were pretreated with 5000 U IFNαB/D for 16 h before infection with TBEV MOI 0.1. Viral titers were determined by focus forming assay at indicated time points (*n* = 6). Primary astrocytes and MEFs were infected with TBEV using MOI 0.1. Expression levels of IFN-β (**c**) and IFNα_2_ (**d**) were determined by qPCR (*n* = 9). Supernatants were collected at the indicated time points, and antiviral activity was determined by VSV-GFP bioassay on MEFs (**e**, *n* = 6) or TBEV-based bioassay on WT and IFNAR^−/−^ astrocytes (**f**, *n* = 12). **g** WT and IFNAR^−/−^ astrocytes were treated with either 5000 U IFNαB/D or virus-inactivated supernatant from WT astrocytes 24 hpi (*n*= ﻿6). TBEV replication was quantified using qPCR and normalized to input viral RNA. Mean values and standard deviations from three independent experiments are shown. *p < 0.05; **p < 0.01; ***p < 0.001

Previous studies have indicated the importance of astrocyte-produced IFN in various viral infections [18, 22, 23]. The observed differences in the impact of IFNAR on astrocytes and MEFs could either be explained by the ability to respond to, or the capacity to induce, IFN following infection. To determine the impact of the type I IFN response in the overall antiviral response, astrocytes and MEFs were pretreated with IFNαB/D [49] Sixteen hours before TBEV infection (Fig. 3a, b). TBEV growth was inhibited to a similar extent showing that both cell-types were able to mount a strong antiviral response upon IFN treatment. Thus, the ability to respond to IFN (Fig. 3a, b) could not explain the lack of control of viral replication in IFNAR^−/−^ MEFs (Fig. 1d, e). To test whether astrocytes and MEFs differ in their capability to induce type I IFNs after infection, IFNβ and IFNα2 mRNAs were quantified by real-time qPCR (Fig. 3c, d). Interestingly, the IFN responses in the two cell types were quite different. Whereas astrocytes induced IFN mRNA after 6 h, the response in MEFs was delayed and IFNβ and IFNα2 mRNAs were detected only after 24 or 48 hpi, respectively. Next, a VSV-GFP-based bioassay on MEFs was applied to quantify secreted IFN, and astrocytes were found to secrete higher levels of IFNs 24 hpi compared to MEFs (Fig. 3e). These data indicate that astrocytes react to viral infection by the induction of IFNs much more rapidly than MEFs.

In order to define the time point after infection at which astrocytes initiate an antiviral response and limit viral spread in astrocytes, a TBEV-based bioassay on astrocytes was developed. Pretreated astrocytes were infected, and the numbers of infected cells were quantified at 48 hpi (Fig. 3f). The supernatants showed an inhibitory effect as early as 6 hpi on WT, but not on IFNAR^−/−^ cells, suggesting a very early type I IFN-mediated antiviral effect.

The antiviral effect of the supernatant was further investigated using qPCR as a more sensitive method of quantifying viral replication. Sixteen hours before TBEV infection, the cells were treated with either supernatant of 24-h TBEV-infected cells or IFNαB/D. The cells were infected with TBEV, and total RNA was extracted 48 hpi to detect TBEV RNA (Fig. 3g). Pretreatment with supernatant of infected cells and 5000 U IFNαB/D had a similar inhibitory effect on TBEV replication in WT astrocytes. However, no difference in viral replication was detected in IFNAR^−/−^ astrocytes treated with or without supernatant, indicating a type I IFN-dependent antiviral effect. These results further suggest that the rapid type I IFN response is responsible for limiting viral infection and replication in astrocytes.

### IFNAR mediates antiviral preparedness in astrocytes

Our data indicate that WT astrocytes are quite resistant to viral infection and that the main effectors in the supernatant from virus-infected astrocytes are type I IFNs. To characterize these responses in more detail, we analyzed transcriptional regulation by RNAseq. Gene expression levels from uninfected IFNAR^−/−^ astrocytes and IFNαB/D-treated, or supernatant-treated, WT astrocytes were compared to untreated WT astrocytes. Fold-change values of at least twofold over the uninfected control and a *q* value of <0.05 were considered indicative of up- or downregulation. Our data yielded 732 upregulated transcripts while 944 were found to be downregulated in IFNAR^−/−^ astrocytes. Treatment of primary astrocytes with IFNαB/D or supernatant led to 634 or 1092 upregulated and 149 or 423 downregulated genes, respectively (Additional file 1: Table S1).

**Fig. 4:**
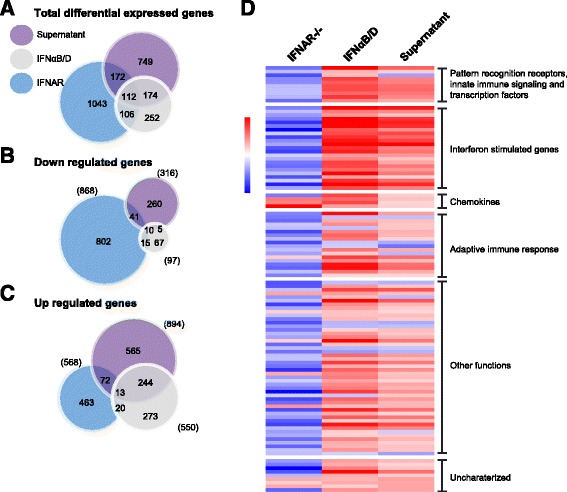
Astrocytes treated with either supernatant or IFNαB/D mount an antiviral response. Primary astrocytes from WT and IFNAR^−/−^ mice were isolated, and WT cells were either mock treated, treated with IFNαB/D, or treated with inactivated supernatant from TBEV-infected astrocytes. Gene expression was analyzed by deep sequencing. Venn diagram of differentially expressed transcripts in IFNAR^−/−^, IFNαB/D, and supernatant-treated astrocytes compared to mock-treated WT astrocytes (fold-change ±2, *q* value <0.05). Number of totally regulated genes (**a**), downregulated (**b**), and upregulated (**c**). **d** Overlap of differentially expressed transcripts under all three conditions (*n* = 112); *red color* indicates an upregulation whereas blue color correlates with a downregulation. Dynamic range in the heat map is 7.16 to −5.99 log2 fold difference compared to WT untreated astrocytes. *Sd* standard deviation (*n* = 3)

The majority of all significantly regulated genes showed a unique pattern of regulation for each sample (Fig. 4a–c). Looking more closely at the differences between astrocytes treated with supernatant or IFNαB/D, relatively few genes were downregulated, and we detected 257 genes upregulated in both samples, out of total 894 upregulated genes in the supernatant and 550 in IFNαB/D (Fig. 4b, c). This indicated that although the main transcriptional response is type I IFN-dependent and overlaps with IFNαB/D treatment, virus infection induces numerous other IFN-independent responses in neighboring, uninfected astrocytes.

Only 112 transcripts were found to be significantly regulated between all three samples compared to WT (Fig. 4a). However, they showed a very different expression profile (Fig. 4d). Interestingly, IFNAR-deficient astrocytes showed lower levels of PRR, innate immune signaling, transcription factors, ISGs, and adaptive immune response compared to untreated WT astrocytes, whereas, IFNαB/D- and supernatant-treated WT astrocytes showed an upregulated profile (Fig. 4d, Additional file 1: Table S2).

### WT astrocytes show higher expression and rapid induction of antiviral ISGs after TBEV infection

**Fig. 5:**
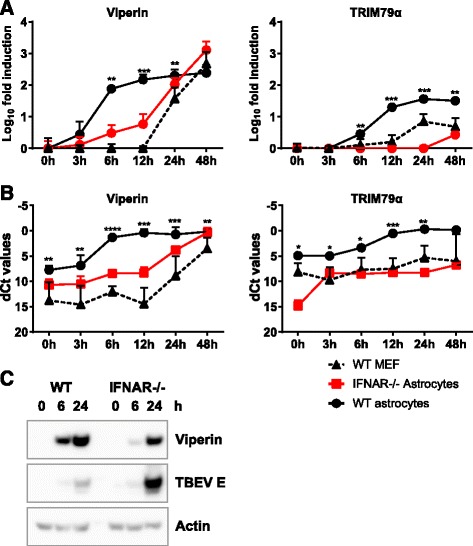
Astrocytes express higher baseline levels and upregulate ISGs faster after TBEV infection compared to MEFs. Primary astrocytes (WT and IFNAR^−/−^) and MEFs were infected with TBEV MOI 0.1, and total cell RNA was extracted at indicated times post infection. Expression levels of viperin and TRIM79α (**a**, **b**) were measured by qPCR analysis, normalized to the cellular GAPDH mRNA. Fold induction over mock (**a**) and dCT values (**b**) are depicted. Mean values and standard deviations from three independent experiments performed in triplicates are shown (*n* = 9). *Asterisk* indicates the significance level between WT astrocytes and MEF. *p < 0.05, **p < 0.01, ***p < 0.001. Protein levels of viperin, TBEV, and actin was detected in TBEV-infected WT and IFNAR^−/−^ astrocytes over time (**c**)

The susceptibility of IFNAR^−/−^ astrocytes to flavivirus infection might be due to lowered basal expression of antiviral ISGs, such as viperin. Together with TRIM79α, viperin has recently been identified as an inhibitor of TBEV in vitro [50, 61]. Viperin has also been shown to have antiviral activity against WNV [62]. Viperin and TRIM79α mRNAs were quantified over time by qPCR in WT and IFNAR^−/−^ astrocytes and WT MEFs (Fig. 5a, b). ISGs were rapidly induced in WT astrocytes at early time points whereas induction was delayed in IFNAR^−/−^ astrocytes and WT MEFs. The basal levels of viperin and TRIM79α were higher in WT astrocytes compared to WT MEFs and IFNAR^−/−^ astrocytes (Fig. 5b) and might contribute to limit initial viral infection. Viperin protein was also strongly induced in WT astrocytes as early as 6 hpi whereas corresponding protein levels were only reached after 24 hpi in IFNAR^−/−^ astrocytes (Fig. 5c). Together, these data show that WT astrocytes express higher basal levels of a subset of antiviral genes and rapidly respond upon viral infection to express antiviral protein that can limit viral infection. MEFs and IFNAR^−/−^ astrocytes show lower basal expression of some antiviral ISGs and a delayed IFN response and ISG induction which is likely to render them more susceptible to viral infection.

### IFN signaling restricts TBEV spread in astrocytes

**Fig. 6:**
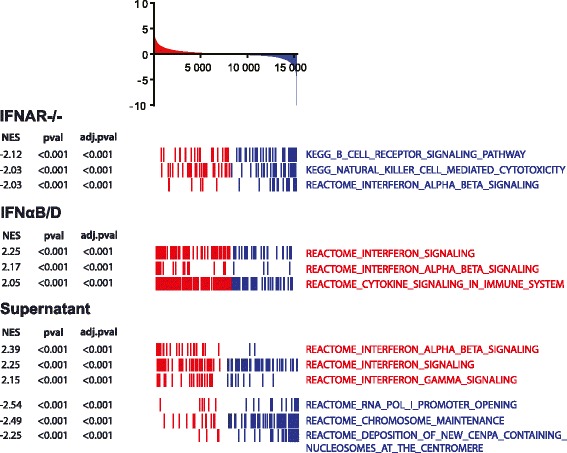
Gene set enrichment analysis of transcriptomic changes. The schematic histogram shows a typical expression profile of the transcriptomic changes after treatment (*y*-axis = fold changes; *x*-axis = one vertical line (stick) represents one transcript; *red*, up; *blue*, down). mRNA transcripts (sticks) and gene sets (names) that are high in IFNAR^−/−^ astrocytes, IFNαB/D and supernatant-treated WT astrocytes, compared to WT astrocytes, are marked as *red*. Genes and gene sets that are downregulated or have a negative normalized enrichment score (NES), respectively, compared to WT astrocytes are blue. *pval* nominal *p* value, *adj.pval* Benjamini-Hochberg adjusted *p* value. An adj.pval <0.05 is regarded significant

**Fig. 7:**
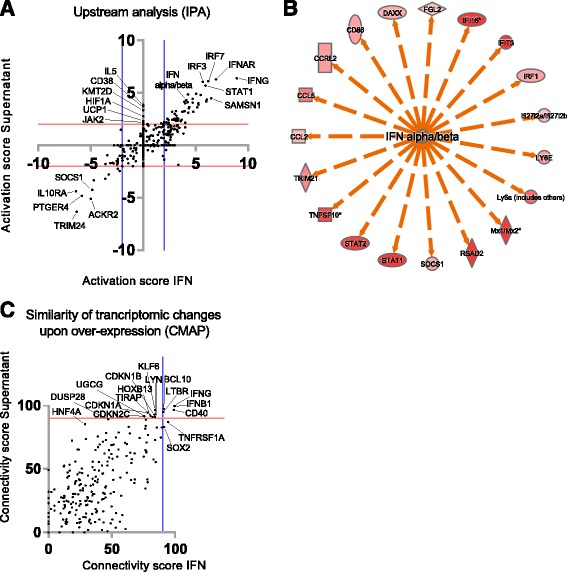
Upstream activators related to treatment with either supernatant or interferon alpha (IFN). **a** Scatter blot of ingenuity pathway analysis (IPA) showing upstream regulators that are predicted to be activated (>2 activation score) or inhibited (<−2 activation score) according to experimental and literature findings. *Y*-axis show upstream regulators predicted from supernatant-treated astrocytes, and *x*-axis show upstream regulators predicted from IFNαB/D-treated astrocytes. Five upstream regulators show an idiosyncratic activation specific for the supernatant-treated cells, aligning the *y*-axis and with an activations score of >2. **b** An example of predicted activators by IPA. The circle of markers is upregulated transcripts (*red*), and the center is a regulator (IFN alpha/beta), predicted to be active based on information (*orange arrows*) in the IPA knowledge-database. Each *black dot* in **a** is representing a similar circle of regulated markers. **c** Genes that, when overexpressed, lead to a similar expression pattern as either supernatant treatment (*y*-axis) or IFNαB/D treatment (*x*-axis) according to the connectivity map database (CMAP). Genes predicted by both treatments are in the *top right corne*r (e.g., IFNG and IFNB1) while effects exclusive to supernatant treatment are high (>90) on the *y*-axis and low (<90) on the *x*-axis

Gene set enrichment analysis was used to identify classes of genes overrepresented in the dataset. IFNAR^−/−^ cells showed a negative enrichment compared to WT in immune pathways and interferon signaling, while the positively enriched gene sets were below our cut-off adjusted *p* value < 0.05 (Fig. 6). Both IFNαB/D and supernatant treatment lead to enrichment in “interferon signaling” and “interferon alpha beta signaling” (Fig. 6). Differentially expressed genes between WT and the three samples were analyzed using IPA to uncover predicted activation of canonical pathways and disease functions. Pathways involved in viral recognition and antiviral signaling as well as functions involved in viability of leukocytes and antiviral and inflammatory responses were found to be downregulated in IFNAR^−/−^ compared to WT astrocytes (Additional file 1: Tables S3 and S4), which can explain why the IFNAR^−/−^ cells responded poorly after viral infection. IPA analysis revealed activation of pathways involved in innate immune signaling and viral recognition, antiviral and immune response after treatment with IFNαB/D and supernatant (Additional file 1: Table S3 and S4). To extend our analyses and investigate what might be responsible for these transcriptomic changes, with focus on the difference between the IFNαB/D and the supernatant treatment, we performed an IPA upstream analysis, and a Connectivity Map (CMAP) analysis. Both of these analyses evaluate the relationship(s) that might have led to the pattern of transcript regulation that we found after treatment. In Fig. 7a, we have plotted all upstream regulators that are predicted by the IPA analysis to be either activated or inhibited after IFNαB/D or supernatant treatment. Upstream regulators that are predicted to be activated after both treatments include several interferon regulated factors (IRF3, IRF7, STAT1) as well as IFNAR and IFNγ as the most activated. Each dot in Fig. 7a represents a regulator e.g., (IFN alpha/beta) with a circle of markers of upregulated transcripts found in the dataset (Fig. 7b). Interestingly, five upstream regulators were predicted to be activated specifically after supernatant treatment (IL5, CD38, KMT2D, HIF1A, UCP1, and JAK2; Fig. 7a, *y*-axis >2). The CMAP database is an orthogonal bioinformatics tool that correlates specific gene-overexpression with their associated transcriptomic changes. Our transcriptional changes after IFNαB/D or supernatant treatments were compared to the CMAP database and the connectivity scores are compared to each other in Fig. 7c, and top scoring genes included IFNB1, IFNγ, and CD40. IFNγ came up in both gene set enrichment analyses, IPA and CMAP (Figs. 6, 7a, b), suggesting that TBEV induces IFNγ after infection in astrocytes. However, no differences in IFNγ mRNA expression levels were detected after infection in astrocytes (data not shown).

With this comprehensive bioinformatics analysis, we can show that the main gene sets enriched after supernatant treatment are IFN signaling gene sets very similar to the IFNαB/D treatment (Fig. 6), overall indicating that IFNs are the main response induced after infection. We show that IFNAR^−/−^ astrocytes not only express lower levels of key antiviral ISGs (Fig. 5) but also express lower levels of ISGs, PRRs, innate immune signaling, and transcription factors generally (Fig. 4d and Additional file 1: Table S2), which probably contributes to the susceptibility of these cells (Figs. 1 and 2).

**Fig. 8:**
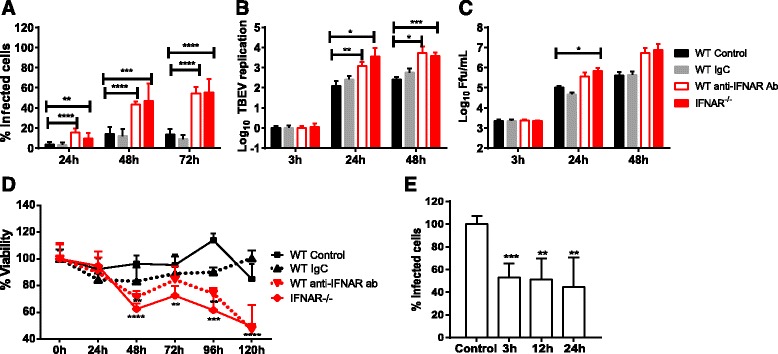
Type I IFN response in astrocytes protects against viral spread of TBEV and cell death. Primary astrocytes isolated from WT and IFNAR^−/−^ mice were infected with TBEV MOI of 0.1 and viral growth was determined at indicated time points. Two hours before infection, cells were treated with anti-IFNAR antibody or control IgG (IgC). Number of infected cells were measured by immunofluorescent staining of TBEV E antigen and DAPI (**a**, *n* = 8). Quantification of virus RNA determined by qPCR analysis (**b**, *n* = 6). Viral titers in cell culture supernatants determined by focus forming assay (**c**, *n* = 6). Cell viability after infection was measured using a resazurine viability assay (**d**, *n* = 8). Supernatants from TBEV-infected astrocytes were collected at the indicated time points and inactivated. Primary neurons were pretreated with the supernatants followed by TBEV infection, and viral infection was determined 48 h post infection by immunofluorescent staining of TBEV E antigen and DAPI (**e**, *n* = 6). Mean values and standard deviations from three independent experiments are shown. *p < 0.05; **p < 0.01; ***p < 0.001; ****p < 0.0001

Astrocytes resistance to viral infection might be due to high basal expression of antiviral genes, fast response via IFNAR signaling, or both. In order to distinguish between these possibilities, monoclonal antibodies were used to block IFNAR signaling in WT astrocytes [63] (Fig. 8a–c). Interestingly, the numbers of TBEV-infected cells and TBEV replication were increased to levels similar to those measured in IFNAR^−/−^ cells. This indicated that response to type I IFN is more important for restricting viral growth than the basal expression level of antiviral ISGs. Previous studies have shown that astrocytes are resistant to TBEV-induced cytopathic effects [34, 35]. We can now show that the viability of WT astrocytes after TBEV infection is dependent on the type 1 IFN response, since IFNAR^−/−^, as well as WT astrocytes treated with an IFNAR-specific antibody, rendered them susceptible to TBEV-induced cytopathic effect 48 hpi (Fig. 8d).

Neurons are the main target of neurotropic flavivirus infection; however, it is not clear if and how astrocytes influence viral growth in neurons. Therefore, supernatants from infected astrocytes were collected at different time points post infection and inactivated. Primary neurons were treated with supernatants before TBEV infection. The number of infected neurons decreased already when pretreated with supernatants from astrocytes infected for 3 h (Fig. 8e). These data indicate that astrocytes can mediate a very fast antiviral effect on neurons already 3 h post infection.

Here, we show that astrocytes are in an antiviral state and respond quickly to flavivirus infection by upregulating type 1 IFNs which limits neurotropic flavivirus spread. Secreted IFNs control viral replication by upregulating several innate immune pathways and induce cell survival. Our data suggest that although neurons seem to represent the main target for neurotropic flavivirus infection in vivo [64], astrocytes are likely to play an important role in responding to infection, amplifying the type I IFN response and limiting viral spread in both astrocytes and neurons.

## Discussion

Previous work has indicated that a local type I IFN response is indispensable for the control of viral replication in the CNS after flavivirus infection [11]. TBEV preferentially replicates in neurons [11, 33], but why astrocytes are less susceptible to TBEV infection remains unclear. In the current study, we showed that astrocytes are initially infected by TBEV but showed strong inhibition of viral spread. Astrocytes showed a higher basal level of ISG expression which enables the cells to rapidly respond with type I IFN production. Thus, astrocytes induce an early and strong antiviral response that limits viral spread in neurons and thereby plays a specific role in the innate antiviral defense in the CNS.

Local type I IFN production is critical to limit viral spread within the CNS [65] whereas CNS deficiency in IFNAR increases the susceptibility of lethal virus infection [13, 17, 18, 66–68]. Using the TBEV model LGTV, we have previously demonstrated the impact of locally produced type I IFN response within the CNS [11]; however, it is not clear which cells produce type I IFN in the CNS during TBEV infection. Astrocytes have been shown to produce an array of innate inflammatory mediators upon stimulation using polyinosinic:polycytidylic acid, lipopolysaccharide, and toll-like receptor (TLR)-7 and TLR-9 agonists [69–72]. Studies have shown that astrocytes are the main producers of type I IFN within the CNS during VSV and La Cross virus infections [18, 23]. Recent studies have shown that TBEV and WNV can infect astrocytes in vitro but fail to spread from cell to cell. However, what prevents viral spread in astrocytes is not known [34, 36]. Our recent results show that neurons are the main target of LGTV infection; however, a small number of astrocytes also become infected. During infection, astrocytes show an activated phenotype in vivo and more cells become infected after LGTV infection in IPS-1-deficient mice [33], indicating that type I IFN response might contribute to the restricted growth of virus in astrocytes.

In this study, we show that, although primary astrocytes are infected in a comparable manner to MEF initially, viral replication and spread is dramatically inhibited, indicating that astrocytes are abortively infected with neurotropic flavivirus similar to La Crosse virus infections [73]. This phenotype is dependent on IFNAR expression, since viral replication is uncontrolled in IFNAR-deficient astrocytes. Consistent with previous results for WNV, we now show for all three subtypes of TBEV, JEV, and ZIKV that the rapidly produced type I IFN in astrocytes after neurotropic flavivirus infection limits the viral spread and prevent virus-induced killing of the cells.

Weak IFN signals, transmitted independently of viral infection, could be crucial for predisposing cells to amplify their IFN production in response to viral infection and enhance their response to other cytokines [74, 75]. Intriguingly, WT astrocytes were in an antiviral state with higher levels of ISGs compared to MEFs, and these were rapidly induced to even higher levels after TBEV infection. Because no difference in basal levels of IFNβ or IFNα2 was observed between MEFs and WT astrocytes (data not shown), a different mechanism compared to IFN priming may exist [76]. However, it seems that the responsiveness to viral infection is determined by basal levels of innate immune components. We have reported previously that TBEV is able to delay IFNβ induction by hiding its dsRNA within replication vesicles in A549 cells [42, 77] and a similar observation was made in the MEFs in this study. No delay in type I IFN induction was observed in astrocytes, clearly indicating that if certain antiviral factors are present in the cell at a basal level the virus is unable to delay the IFN response. High basal expression of ISGs have also been linked to viral resistance to influenza A infection in bronchial epithelial cells [78] and contribute to neuronal tropism of WNV [19]. Furthermore, we previously showed that viperin is a strong inhibitor of TBEV infection [50]. Therefore, the increased basal expression of some key ISGs could contribute to lower flavivirus replication in astrocytes. However, the basal expression was not enough by itself to restrict TBEV replication or spread as neutralization of IFNAR with antibodies in WT astrocytes rendered the WT cells susceptible to TBEV infection. Virus infection has been shown to directly induce antiviral ISGs independently of IFN signaling [79]; however, in the case of TBEV infection of astrocytes, this response does not seem to inhibit TBEV growth.

Although IFNAR^−/−^ astrocytes did induce ISGs at the mRNA level and viperin at the protein level, the kinetics were delayed compared to WT astrocytes indicating the importance of a rapid IFN response in order to control TBEV infection. To further characterize what makes the bystander cells resistant to flavivirus infection, astrocytes were treated with either inactivated supernatant from virus-infected cells or with IFNαB/D followed by RNAseq. The RNAseq revealed that IFN signaling was the most upregulated pathway in supernatant-treated cells suggesting the IFN might be the most important signaling molecule produced by astrocytes upon TBEV infection. This is also true for other viruses, as increased expression of IFN signaling molecules and ISGs was observed in human astrocytes infected with Junin virus [52].

Comparison of the differently expressed genes among WT, IFNAR^−/−^ astrocytes, supernatant or IFNαB/D-treated WT astrocytes showed an overlap of 112 transcripts, and these transcripts might be of particular importance as most of them were downregulated in IFNAR^−/−^ astrocytes, which were highly susceptible to TBEV infection, whereas they were upregulated in supernatant- and IFNαB/D-treated cells, which were resistant to the infection. Several antiviral ISGs were identified among the overlap such as viperin and TRIM79α, which have been identified as inhibitors of TBEV [50, 61]. ISG15, viperin, and Oas1b, which were found to be downregulated in IFNAR^−/−^ astrocytes and upregulated after treatment with IFNαB/D and supernatant, have previously been identified as inhibitors of WNV and could thus contribute to antiviral response against WNV in astrocytes [19, 62, 80, 81].

Predicting upstream regulators responsible for the different expression patterns in IFNAR^−/−^-, supernatant-, and IFNαB/D-treated astrocytes identified type I IFN as well as IFN signaling molecules to have the highest activation scores. Similar findings were found in a previous study where IFN signaling and antiviral mediators were among the most upregulated pathways and genes in WNV-infected mice brains [51]. IFNγ-STAT1-IRF-1 signaling cascade was predicted as an upstream regulator both in supernatant and IFNαB/D-treated astrocytes. Although IFNγ transcripts were not detected in TBEV infected, astrocytes IRF-1 could be directly induced by virus infection and could be responsible for the induction of the overlap between inducible genes among the type I and II IFNs [79, 82–84]. Taken together, the RNAseq confirms the potent IFN response of the astrocytes and identifies a subset of genes as key players in determining the outcome of TBEV infection in astrocytes.

Previous studies have revealed that although TBEV infects astrocytes, viral infection did not affect the viability of the cells [34, 35]. Similar findings have been observed with other viral infections such as WNV, JEV, and Junin virus whereas infection with Venezuelan equine encephalitis virus infection induced cell death in cultured astrocytes [31, 37, 39, 85, 86]. However, the mechanism underlying the resistance to virus-induced cell death in astrocytes is not well understood. Here, we show that the type I IFN response prevents TBEV-mediated cytopathic effect. Blocking of IFNAR using antibodies as well as IFNAR knockout induced TBEV-mediated cell death of astrocytes whereas the WT control remained unaffected.

## Conclusions

Our results show that astrocytes mount a rapid type I IFN response upon flavivirus infection that inhibits viral spread and replication. The IFN response also protects astrocytes from virus-induced cell death. We further propose that astrocytes are important IFN producers in flavivirus infection, which can sense the viral infection and then mediate a local IFN response within the CNS, which could protect not only astrocytes but also other CNS resident cells.

## Additional file

Additional file 1: Supplemental Table S1, Table S2, Table S3, and Table S4. (DOC 210 kb)

## Abbreviations

CMAP: Connectivity Map
dsRNA: Double-stranded RNA
GFAP: Glial fibrillary acidic protein
GSEA: Gene set enrichment analysis
hpi: Hours post infection
IFN: Interferon
IFNAR: Interferon alpha receptor
IPA: Ingenuity Pathway Analysis
IPS-1: Interferon-beta promoter stimulator 1
IRF: Interferon regulatory factor
ISG: Interferon-stimulated gene
ISGF3: Interferon-stimulated gene factor-3
JEV: Japanese encephalitis virus
LGTV: Langat virus
MEF: Mouse embryonic fibroblast
NES: Normalized enrichment score
PRR: Pattern recognition receptor
qPCR: Quantitative reverse transcription PCR
RNAseq: RNA sequencing
STAT: Signal transducer and activator of transcription
TBEV: Tick-borne encephalitis virus
TRIM79α: Tripartite motif 79 α
VSV: Vesicular stomatitis virus
WNV: West Nile virus
WT: Wild-type
ZIKV: Zika virus
β-PL: Beta propiolactone
