# Supplementary material for: Fast type I interferon response protects astrocytes from flavivirus infection and virus-induced cytopathic effects
Source: J Neuroinflammation. 2016 Oct 24;13:277. doi: 10.1186/s12974-016-0748-7 (PMC5078952; doi:10.1186/s12974-016-0748-7)
Supplement: Additional file 1: — Supplemental Table S1, Table S2, Table S3, and Table S4. (DOC 210 kb) [file 12974_2016_748_MOESM1_ESM.doc]

Additional file 1: Table S1.

|  |  | Number of differentially expressed genes (P < 0,05)* | | | | |
| --- | --- | --- | --- | --- | --- | --- |
|  |  | Total | >50X | 10-50X | 5-10X | 2-5X |
| **IFNAR-/-** | Upregulated | 732 | 12 | 46 | 107 | 567 |
|  | Downregulated | 944 | 2 | 100 | 231 | 611 |
| **Supernatant** | Upregulated | 1092 | 1 | 57 | 142 | 892 |
|  | Downregulated | 423 | 0 | 4 | 30 | 389 |
| **IFNαB/D** | Upregulated | 634 | 33 | 103 | 108 | 390 |
|  | Downregulated | 149 | 0 | 0 | 2 | 147 |

* WT astrocytes were treated with either inactivated supernatant from TBEV infected cells or 5000U of IFNαB/D. RNA sequencing was performed on IFNAR^-/-^ and WT astrocytes treated with supernatant or IFN αB/D. Differential gene expression was compared to untreated WT astrocytes and calculated by GATC biotech, statistical analysis were done using cufflinks.

Additional file 1: Table S2. /

| **Pattern recognition receptors, innate immune signaling and transcription factors** | | | | |  |  | **Other function** | | | |
| --- | --- | --- | --- | --- | --- | --- | --- | --- | --- | --- |
|  | | **IFNAR-/-** | **IFNαB/D** | **SUP** |  |  |  | **IFNAR-/-** | **IFNαB/D** | **SUP** |
| Irf7 | | -3,19047 | 6,00598 | 3,66861 |  |  | Adgb | -3,92427 | -1,4771 | -1,75034 |
| Irf9,Rnf31 | | -1,49029 | 1,79094 | 1,18624 |  |  | AI607873 | -3,78064 | 2,45727 | 1,4092 |
| Cd180 | | -3,99244 | 1,55538 | -2,10298 |  |  | Apol9a | -1,34474 | 4,40189 | 4,03018 |
| Ddx60 | | -2,61879 | 4,493 | 2,91394 |  |  | Btc | 2,12049 | 2,32902 | 1,58834 |
| Dhx58 | | -2,5513 | 3,7954 | 2,94436 |  |  | Ch25h | -2,34679 | 1,61389 | -1,54928 |
| Nlrc5 | | -1,51443 | 4,17224 | 3,40178 |  |  | Cmpk2 | -1,45169 | 6,01081 | 4,01955 |
| Stat1 | | -1,42247 | 4,23456 | 3,53723 |  |  | Epsti1 | -4,38101 | 2,67356 | 1,70412 |
| Stat2 | | -1,35535 | 3,50309 | 3,19593 |  |  | Fam46a | -1,85349 | 2,17799 | 1,24341 |
| Gbp3 | | -1,6144 | 4,67036 | 2,80129 |  |  | Filip1 | 1,20378 | 1,43629 | 1,00383 |
| Gbp4,Gbp9 | | -3,38422 | 2,86607 | 1,99389 |  |  | Gpr35 | -1,37965 | 1,43699 | 1,60378 |
| **Chemokines** | | | | |  |  | Hk3 | -2,89747 | 1,66501 | 1,28727 |
|  |  |  |  |  |  |  | Hpgd | -3,96294 | -1,64504 | -1,85166 |
|  | **IFNAR-/-** | | **IFNαB/D** | **SUP** |  |  | Kcnj2 | -2,24696 | -1,52714 | -1,37596 |
| Ccl12 | -3,00995 | | 4,26264 | 1,21128 |  |  | Klra2 | -3,0383 | 1,12663 | 1,58108 |
| Ccl2 | 3,13088 | | 3,56426 | 1,46161 |  |  | Lgals3bp | -1,62363 | 2,38282 | 1,30966 |
| Ccl7 | 2,8625 | | 4,28936 | 1,03664 |  |  | Lgals9 | -1,98854 | 2,99683 | 1,84121 |
| Cxcl1 | 5,98699 | | 2,2169 | 1,14297 |  |  | Mmp13 | 1,14449 | 7,07635 | 3,16375 |
| **Interferon stimulated genes** | | | | |  |  | Ms4a6d | -3,36197 | 1,44521 | 1,06715 |
|  |  |  |  |  |  |  | Nlrp10 | -1,49027 | -1,58602 | -1,26232 |
|  | | **IFNAR-/-** | **IFNαB/D** | **SUP** |  |  | Nxpe5 | -3,17532 | -2,28564 | -1,68553 |
| Rsad2 | | -2,85943 | 6,79015 | 5,41625 |  |  | Parp14 | -1,40945 | 3,48643 | 2,38607 |
| Trim30d | | -1,39767 | 3,02538 | 2,73984 |  |  | Pvrl4 | -1,30649 | 1,79872 | 1,54317 |
| Bst2 | | -2,28184 | 2,47402 | 1,51256 |  |  | Pyhin1 | -2,80414 | 4,53299 | 3,12366 |
| Isg15 | | -2,97698 | 5,84555 | 4,68185 |  |  | Rnase6 | -3,19638 | 1,92816 | 1,10199 |
| Mx1 | | -4,63969 | 7,02507 | 5,21965 |  |  | Rtp4 | -4,70172 | 3,68971 | 2,71843 |
| Mx2 | | -3,01407 | 5,89011 | 4,92629 |  |  | Rxrg | 2,03353 | 2,80846 | 1,42411 |
| Oas1a | | -5,86868 | 3,95629 | 2,69178 |  |  | Slamf8 | -2,16413 | 3,07515 | 1,62359 |
| Oas1b | | -1,11287 | 4,13644 | 4,29695 |  |  | Slc2a6 | -1,06537 | 2,16591 | 1,07713 |
| Oas2 | | -4,3076 | 4,88309 | 3,5317 |  |  | Slfn10-ps | -2,185 | 2,76586 | 2,10354 |
| Oas3 | | -2,64062 | 5,41787 | 4,42599 |  |  | Slfn2 | -2,9062 | 3,06377 | 1,77768 |
| Oasl1 | | -1,96508 | 7,03746 | 5,82083 |  |  | Slfn5 | -5,57483 | 4,53245 | 2,60187 |
| Oasl2 | | -3,88192 | 4,73725 | 3,01634 |  |  | Snai3 | -1,00223 | -1,26663 | 1,31034 |
| Slfn8 | | -4,37386 | 5,28623 | 3,27663 |  |  | Sp100 | -4,17575 | 3,63034 | 2,38504 |
| Trim25 | | -1,03611 | 2,36558 | 2,44283 |  |  | Sp110 | -3,27064 | 2,87647 | 1,73015 |
| Trim14 | | -1,79095 | 2,88418 | 1,44384 |  |  | Zfp536 | 2,54123 | 1,54858 | 1,83903 |
| AW112010 | | -3,08911 | 4,40297 | 2,06968 |  |  | Zbp1 | -4,02697 | 5,17273 | 3,41362 |
| Ifi203,Mndal | | -3,33229 | 4,3307 | 3,20239 |  |  | Xdh | -1,71583 | 1,95412 | 1,00443 |
| Ifi204 | | -2,50414 | 3,53925 | 2,47221 |  |  | Xaf1 | -2,22918 | 3,88952 | 3,23091 |
| Ifi205 | | -1,51305 | 6,04434 | 3,23624 |  |  | Vnn3 | -3,72119 | 1,16906 | 1,56886 |
| Ifi27l2a | | -4,02169 | 2,12794 | 1,09284 |  |  | Usp18 | -4,22781 | 5,40262 | 4,20815 |
| Igtp,Irgm2 | | -1,06508 | 4,8532 | 3,53231 |  |  | Trim30a | -1,50235 | 3,43115 | 2,84232 |
| Iigp1 | | -4,98585 | 7,16402 | 5,02567 |  |  | Tnf | -2,20477 | 1,20035 | -1,08812 |
| Mnda | | -1,90404 | 4,22066 | 2,94685 |  |  | Tmem221 | -1,80692 | 1,07698 | 1,32596 |
| **Uncharacterized** | | | | |  |  | Themis2 | -3,31663 | 2,86251 | 1,70894 |
|  |  |  |  |  |  |  | Tgm2 | -1,72584 | 1,91637 | 1,19936 |
|  | **IFNAR-/-** | | **IFNαB/D** | **SUP** |  |  | Tagap | -3,50231 | 1,82019 | 1,46149 |
| Gm11425 | -2,26999 | | 2,30316 | 1,6855 |  |  | Stk32c | -1,31602 | 2,48311 | 1,38998 |
| Gm12185 | -2,98979 | | 1,93105 | 1,03838 |  |  | Sprr1a | -1,46713 | -1,059 | -2,07462 |
| Gm12185,Gm5431 | -5,99346 | | 3,00354 | 1,42581 |  |  |  |  |  |  |
| Gm12250 | -3,93426 | | 5,74712 | 3,35429 |  |  |  |  |  |  |
| Gm20412 | -1,6512 | | 1,9315 | 1,23098 |  |  |  |  |  |  |
| Gm20431 | 1,95216 | | 1,72957 | 2,20876 |  |  |  |  |  |  |
| Gm26547 | 1,18728 | | 2,02192 | 2,0328 |  |  |  |  |  |  |
| Gm6277 | 1,9813 | | 1,18551 | 2,15995 |  |  |  |  |  |  |
| Gm8995 | -3,58217 | | 2,61054 | 2,13167 |  |  |  |  |  |  |

**Overlap of significant upregulated genes compared to WT astrocytes among all three conditions, IFNAR^-/-^, IFNαB/D treatment and supernatant treatment.** Negative numbers represent fold downregulated and positive number fold upregulated genes.

Additional file 1: Table S3.

| **Pathway differentially regulated in IFNAR-/-** | **Z-score** | **p-value** | **Ratio** |
| --- | --- | --- | --- |
| TREM1 signaling | -4.536 | 2.152E-11 | 0.412 |
| Role of NFAT in regulating the immune response | -4.315 | 0.001741 | 0.179 |
| Role of PRR in recognition of Bacteria and Virus | -4.2 | 3.311E-11 | 0.325 |
| NFKb signaling | -3.656 | 0.00009462 | 0.201 |
| FCy phagocytosis in macrophages and monocytes | -3.411 | 0.00007177 | 0.244 |
| FCy phagocytosis in macrophages and monocytes | -3.411 | 0.00007177 | 0.244 |
| Activation of NFkb by viruses | -2.828 | 0.00008790 | 0.26 |
| Toll-like receptor signaling | -2.714 | 0.001230 | 0.232 |
| IL-8 signaling | -2.646 | 0.006471 | 0.164 |
| Activation of IRFs by cytosolic PRRs | -2.496 | 0.001667 | 0.25 |
| Production of NO and ROS by macrophages | -2.401 | 0.00001282 | 0.213 |
| CD28 signaling in T-helper cells | -2.357 | 0.0036307 | 0.189 |
| IL-6 signaling | -2.065 | 0.009484 | 0.177 |

| **Pathway differentially regulated after supernatant treatment** | **Z-score** | **p-value** | **Ratio** |
| --- | --- | --- | --- |
| Role of PRR in recognition of Bacteria and Virus | 3 | 0.0006209 | 0.1970 |
| NFKB Signaling | 2.982 | 0.2143 | 0.116 |
| Retinoic acid Mediated apoptosis signaling | 2.828 | 0.02786 | 0.196 |
| Interferon signaling | 2.53 | 6.095E-06 | 0.414 |
| Activation of NFkb by viruses | 2.333 | 0.2594 | 0.116 |
| Toll-like receptor signaling | 2.121 | 0.2094 | 0.13 |
| Eicosanoid signaling | 2 | 0.01164 | 0.197 |
| eNOS signaling | 2 | 0.01999 | 0.153 |
| VDR/RXR activation | 2 | 0.03258 | 0.1687 |
| Role of RIG1 like receptors in innate immunity | 1.633 | 0.08913 | 0.182 |
| Dopamine-DARPP3 Feedback in cAMP signaling | 1.606 | 0.007464 | 0.159 |
| Activation of IRFs by cytosolic PRRs | 1.5 | 0.00001641 | 0.308 |
| TREM1 signaling | 1.387 | 0.01122 | 0.191 |
| Antioxidant action of Vitamin C | -1.897 | 0.03251 | 0.16 |

| **Pathway differentially regulated after IFNαB/D treatment** | **Z-score** | **p-value** | **Ratio** |
| --- | --- | --- | --- |
| TREM1 signaling | 4.025 | 6.714E-11 | 0.294 |
| Role of PRR in recognition of Bacteria and Virus | 3.618 | 5.636E-10 | 0.214 |
| Role of NFAT in regulating the immune response | 3.317 | 0.0004955 | 0.117 |
| NFKB Signaling | 3.12 | 2.249E-06 | 0.146 |
| CD28 signaling in T-helper cells | 2.826 | 0.0004227 | 0.135 |
| Toll-like receptor signaling | 2.53 | 0.0004134 | 0.174 |
| Activation of NFkb by viruses | 2.53 | 0.003350 | 0.137 |
| VDR/RXR activation | 2.236 | 0.19588 | 0.077 |
| Interferon signaling | 2.121 | 7.943E-07 | 0.345 |
| Retinoic acid Mediated apoptosis signaling | 1.89 | 0.00007430 | 0.217 |
| Role of RIG1 like receptors in innate immunity | 1.89 | 0.00002477 | 0.273 |
| Activation of IRFs by cytosolic PRRs | 1.698 | 2.838E-10 | 0.327 |

**Ingenuity pathway analysis of differentially expressed transcripts in astrocytes.** IPA was performed on differentially expressed transcripts. Activation Z-values were used to determine activation of pathways (-2 ≥ Z = significant inhibition, 2 ≤ Z significant activation), p-values were calculated using right-tailed Fisher’s exact representing the significance for the overlap between dataset and pathway. Ratio corresponds to the number of differentially expressed genes divided by the total number of genes within that pathway.

Additional file 1: Table S4.

| **IFNAR-/- astrocytes** |  |  |  |
| --- | --- | --- | --- |
| **Disease/Function** | **Z-score** | **p-value** | **#molecules** |
| Immune response of cells | -3.955 | 1.73E-25 | 118 |
| Antimicrobial response | -3.667 | 9.54E-16 | 54 |
| Phagocytosis | -3.549 | 1.38E-20 | 72 |
| Antiviral response | -3.529 | 7.35E-11 | 28 |
| Cell viability of leukocytes | -2.941 | 4.00E-7 | 43 |
| Inflammatory response | -2.563 | 2.32E-38 | 177 |
| Immune response of macrophages | -2.463 | 5.86E-11 | 38 |
| Infection of mammalian | 3.699 | 3.48E-28 | 120 |
| Organismal death | 2.278 | 8.01E-20 | 459 |
| Viral infections | 1.8 | 8.99E-16 | 76 |

| **Supernatant treated WT astrocytes** |  |  |  |
| --- | --- | --- | --- |
| **Disease/Function** | **Z-value** | **p-value** | **#Molecules** |
| Antiviral response | 1.937 | 1.73E11 | 28 |
| Immune response of cells | 1.913 | 6.11E6 | 69 |
| Inflammatory response of cells | 1.854 | 6.01E5 | 90 |
| Antimicrobial response | 1.555 | 5.05E9 | 47 |
| Infection by RNA virus | -1.313 | 2.66E4 | 15 |
| Viral life cycle | -2.63 | 5.86E4 | 7 |
| Mortality | -3.035 | 3.26E5 | 23 |
| Infection of mammalian | -3.516 | 6.46E10 | 79 |
| Viral infection | -3.585 | 3.31E10 | 62 |
| Organismal Death | -5.754 | 1.64E6 | 367 |

| **IFNαB/D treated WT astrocytes** |  |  |  |
| --- | --- | --- | --- |
| **Disease/Function** | **Z-value** | **p-value** | **#Molecules** |
| Inflammatory response of cells | 3.823 | 1.13E14 | 80 |
| Immune response of cells | 3.429 | 1.57E15 | 64 |
| Antimicrobial response | 2.764 | 3.55E-18 | 41 |
| Antiviral response | 2.578 | 2.49E15 | 25 |
| Viral life cycle | -2.63 | 9.66E6 | 7 |
| Infection by RNA virus | -2.67 | 1.92E9 | 17 |
| Mortality | -3.191 | 7.77E7 | 23 |
| Viral infection | -4.422 | 4.09E-33 | 74 |
| Infection of mammalian | -4.755 | 3.55E-33 | 89 |

**Ingenuity pathway analysis (IPA) of differentially expressed transcripts in astrocytes.** Activation Z-values are indicative of directionality and activity: -2 ≥ Z = significant inhibition, 2 ≤ Z significant activation; p-values were calculated using a right-tailed Fisher’s exact test representing the significance of the overlap between dataset and disease/function. Number (#) of molecules are those that we map from our dataset to a particular disease/function in the IPA database.
